# Δ-Machine Learning to Elevate DFT-Based Potentials and a Force Field to the CCSD(*T*) Level Illustrated for Ethanol

**DOI:** 10.1021/acs.jctc.4c00977

**Published:** 2024-10-03

**Authors:** Apurba Nandi, Priyanka Pandey, Paul L. Houston, Chen Qu, Qi Yu, Riccardo Conte, Alexandre Tkatchenko, Joel M. Bowman

**Affiliations:** † Department of Physics and Materials Science, University of Luxembourg, L-1511 Luxembourg City, Luxembourg; ‡ Department of Chemistry and Cherry L. Emerson Center for Scientific Computation, Emory University, Atlanta, Georgia 30322, United States; § Department of Chemistry and Chemical Biology, Cornell University, Ithaca, New York 14853, United States; ∥ Independent Researcher, Toronto, Ontario M9B0E3, Canada; ⊥ Department of Chemistry, Fudan University, Shanghai 200438, P. R. China; # Dipartimento di Chimica, Università degli Studi di Milano, via Golgi 19, 20133 Milano, Italy; ∇ Department of Chemistry and Biochemistry, Georgia Institute of Technology, Atlanta, Georgia 30332, United States

## Abstract

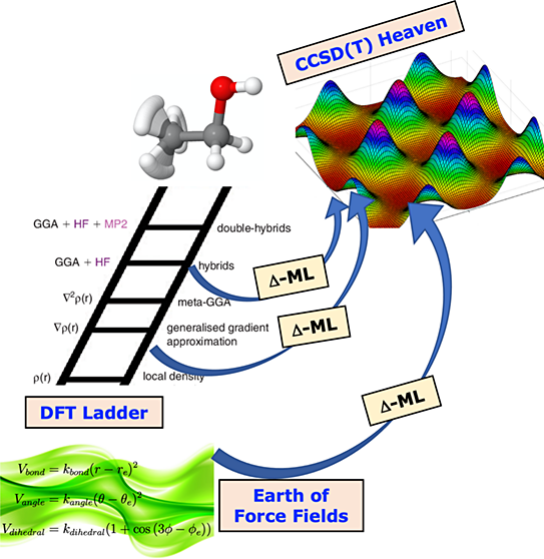

Progress in machine learning has facilitated the development of potentials that offer both the accuracy of first-principles techniques and vast increases in the speed of evaluation. Recently, Δ-machine learning has been used to elevate the quality of a potential energy surface (PES) based on low-level, e.g., density functional theory (DFT) energies and gradients to close to the gold-standard coupled cluster level of accuracy. We have demonstrated the success of this approach for molecules, ranging in size from H_3_O^+^ to 15-atom acetyl-acetone and tropolone. These were all done using the B3LYP functional. Here, we investigate the generality of this approach for the PBE, M06, M06-2X, and PBE0 + MBD functionals, using ethanol as the example molecule. Linear regression with permutationally invariant polynomials is used to fit both low-level and correction PESs. These PESs are employed for standard RMSE analysis for training and test data sets, and then general fidelity tests such as energetics of stationary points, normal-mode frequencies, and torsional potentials are examined. We achieve similar improvements in all cases. Interestingly, we obtained significant improvement over DFT gradients where coupled cluster gradients were not used to correct the low-level PES. Finally, we present some results for correcting a recent molecular mechanics force field for ethanol and comment on the possible generality of this approach.

## Introduction

Developing high-dimensional, ab initio-based potential energy surfaces (PESs) is an active area of theoretical and computational research. Major progress has been made in using and developing machine learning (ML) approaches for PESs with more than four atoms, based on fitting thousands of CCSD(*T*) energies^1−5^ or forces.^6,7^ Some of these ML approaches have used permutationally invariant polynomials (PIPs) or PIPs as inputs to neural network software.^1−5^ Of course, there are numerous other ML methods. It is perhaps of interest and relevance to this paper that the precision of a PIP PES for ethanol was shown to be as good as the best performing ML methods and to be substantially faster (factors of 10 or more)^8^ than all the ML methods considered, i.e., GAP-SOAP,^9^ ANI,^10^ DPMD,^11^ sGDML,^6,7^ PhysNet,^12^ KREG,^13^ and pKREG.^14^ The data set for these comparisons was from the rMD17 database,^15^ which uses 500 K direct dynamics based on the PBE0 functional to obtain energies and forces. The metrics used in the “learning curves” were root-mean-square errors in energies and forces. This followed the standard protocol used earlier to assess many ML methods for potentials.^16^

CCSD(*T*) data sets for larger molecules are rare, owing to the steep scaling of CCSD(*T*) calculations of order ∼ *N,*(7)*N* being the number of basis functions. The potential energy surface for the 10-atom formic acid dimer is one example where PESs have been reported at the CCSD(*T*) level, using PIPs^17^ and later an atom-centered high-dimensional NN.^18^ Complex reactive potentials for 6 and 7-atom chemical reactions, which are fitted to tens of thousands or even hundred thousand CCSD(*T*) energies, have been reported.^19,20^ The PIP-based automated ROBOSURFER software^5^ has been applied to develop a number of complex PESs for 9-atom chemical reactions.^21,22^

Correcting ab initio-based potential energy surfaces has been a long-standing goal of computational chemistry. Of relevance here are approaches that aim to bring a PES, based on a low-level of electronic theory, typically DFT or MP2 theory, to a higher level such as coupled cluster (CC) theory. In consideration of larger molecules and clusters, where high-level methods are prohibitively expensive, the motivation for doing this is clear. “Δ-machine learning” (Δ-ML) is the method of direct relevance to the present paper. This approach seeks to add a correction to a property obtained using an efficient and thus perforce low-level ab initio theory.^6,7,23−25^ A hierarchical Δ-ML method using multiple quantum chemistry methods has been applied to the five-atom CH_3_Cl PES.^26^ In this sense, the approach is related, in spirit at least, to the correction potential approach mentioned above, when the property is the PES. Other, related methods that utilize a high and lower level of electronic structure theory are mentioned in the Discussion section.

We recently applied a Δ-ML approach, originally given for the three-atom F + H_2_,^27^ to larger systems.^28−33^ Additionally, the approach has also been proposed to correct many-body force fields.^34^ In all these examples, the B3LYP functional was used to obtain the low-level PES.

Considering the success of the Δ-ML method with the B3LYP functional,^35,36^ it is both interesting and significant to explore whether this straightforward approach can be extended to other functionals and to molecular mechanics, including “classical” force fields (FFs). There is a vast literature on molecular mechanics force fields, and the reader is directed to a recent and relevant (vide infra) paper that surveys this field.^37^ While these FFs, which are heavily semi or totally empirical, have made an enormous impact in biomolecular simulations, there is strong motivation to progress from these. Broadly put, there are two approaches that can be undertaken. One is to replace these FFs with strictly ML FFs, based on electronic structure energies and forces for the covalent and noncovalent interactions, and sophisticated treatments of long-range interactions. This is a major challenge for an ML method that aims to deal with hundreds of atoms in a single step. A recent example of this approach by Tkatchenko, Müller and co-workers can be found in ref (38). Of course, invoking the “no free lunch” axiom, this approach is far more demanding in computational effort compared to a classical FF. A second approach is to correct a classical force field. There have been several papers along these lines including one from this group aimed at correcting a sophisticated classical FF for water, by correcting the short-range 2-b, 3-b, and even 4-b interactions.^34^ However, while water is essential for life it is not a biomolecule. Other similar approaches, focused on correcting the short-range interactions have also appeared recently.^39^ While these approaches may be less computationally demanding than a full ML approach, they are still far more demanding than biomolecular FFs.

A variation of the second approach, which is our focus, is to continue to use the empirical FF expression for the potential, i.e., harmonic bond stretches and bends, periodic torsional potentials, plus simple 2-b noncovalent interactions and long-range electrostatics, and to add a computationally efficient ML correction. To facilitate the goal of efficiency, the ML correction can be applied to some terms, at least, in the classical FF are corrected using ab initio electronic energies. Recently, Meuwly and co-workers,^37,40^ investigated correcting the CHARMM classical force field for specific examples. An earlier, but still recent, example of this approach used atomic force matching (AFM), using MP2 theory, to determine classical FF intramolecular terms of ethanol plus the 2-b intermolecular interaction between an ethanol and water molecules.^41^ Here, we use this AFM-corrected FF for ethanol to investigate our computationally efficient Δ-ML approach, which substantially improves several key properties of AFM-corrected FF. Most notably, it addresses the harmonic normal-mode frequencies, which are greatly overestimated by this FF for all but the lowest several normal modes.

The paper is organized as follows. A brief review of the Δ-ML approach is provided, along with the essentials of the highly efficient ML linear-regression approach we use with permutationally invariant polynomials. Results and discussion follow, including remarks on the extension of the Δ-ML PIP approach to much larger molecules.

## Theory

### Δ-ML Approach

The Δ-ML approach is given by the equation^28^
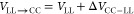
1where *V*_LL→CC_ is the corrected PES, *V*_LL_ is a PES fit to low-level DFT electronic data, and Δ*V*_CC–LL_ is the correction PES based on high-level coupled cluster energies. As shown in ref (28), we use PIPs to represent the PESs on the right-hand side. This might suggest that the cost to evaluate their sum is twice the cost to evaluate *V*_LL_. However, because the correction potential is “small” and more slowly varying over configuration space, relative to *V*_LL_, the PIPs expansion for that PES is much smaller than the one for the *V*_LL_. Thus, the cost of evaluating that term is much less than the cost to evaluate *V*_LL_. If the above were not true, then the Δ-ML would not be a viable approach. We will return to this with specific numbers below for the present application to ethanol. Also, we note that the above equation was given 16 years ago,^27^ and so we rediscovered it.

To investigate the efficacy of the Δ-ML approach, four widely used functionals are employed here, M06^42^ and M06-2X^42,43^ functionals with the 6-311+G(d,p) basis, PBE^44^ with the def2-SVP basis, and PBE0^45,46^ including many-body dispersion (MBD)^47^ with the “intermediate” basis setting.^48^ Additionally, we also replace *V*_LL_ with a classical force field.

**Figure 1:**
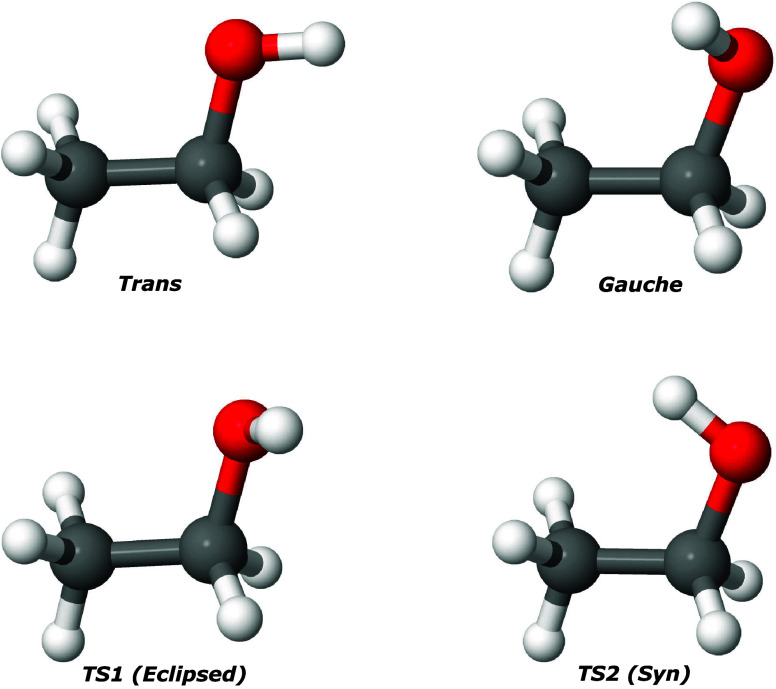
Optimized geometry of *trans* and *gauche* conformers of ethanol and their two isomerization TSs at CCSD(*T*)-F12a/aug-cc-pVDZ level. Reproduced from ref (49). Copyright 2022 American Chemical Society.

**Table 1:** RMS Fitting Error (in cm^–1^) of *V*_LL_ for Training and Test Data Sets

	PBE	M06	M06-2X	B3LYP	PBE0 + MBD
training	45	79	47	40	40
test	56	82	57	51	51

**Table 2:** RMS Fitting Error (in cm^–1^) of Correction PESs Δ*V*_CC–LL_ for Training and Test Data Sets

	PBE	M06	M06-2X	B3LYP	PBE0 + MBD
training	67	53	32	28	26
test	90	61	40	30	30

**Figure 2:**
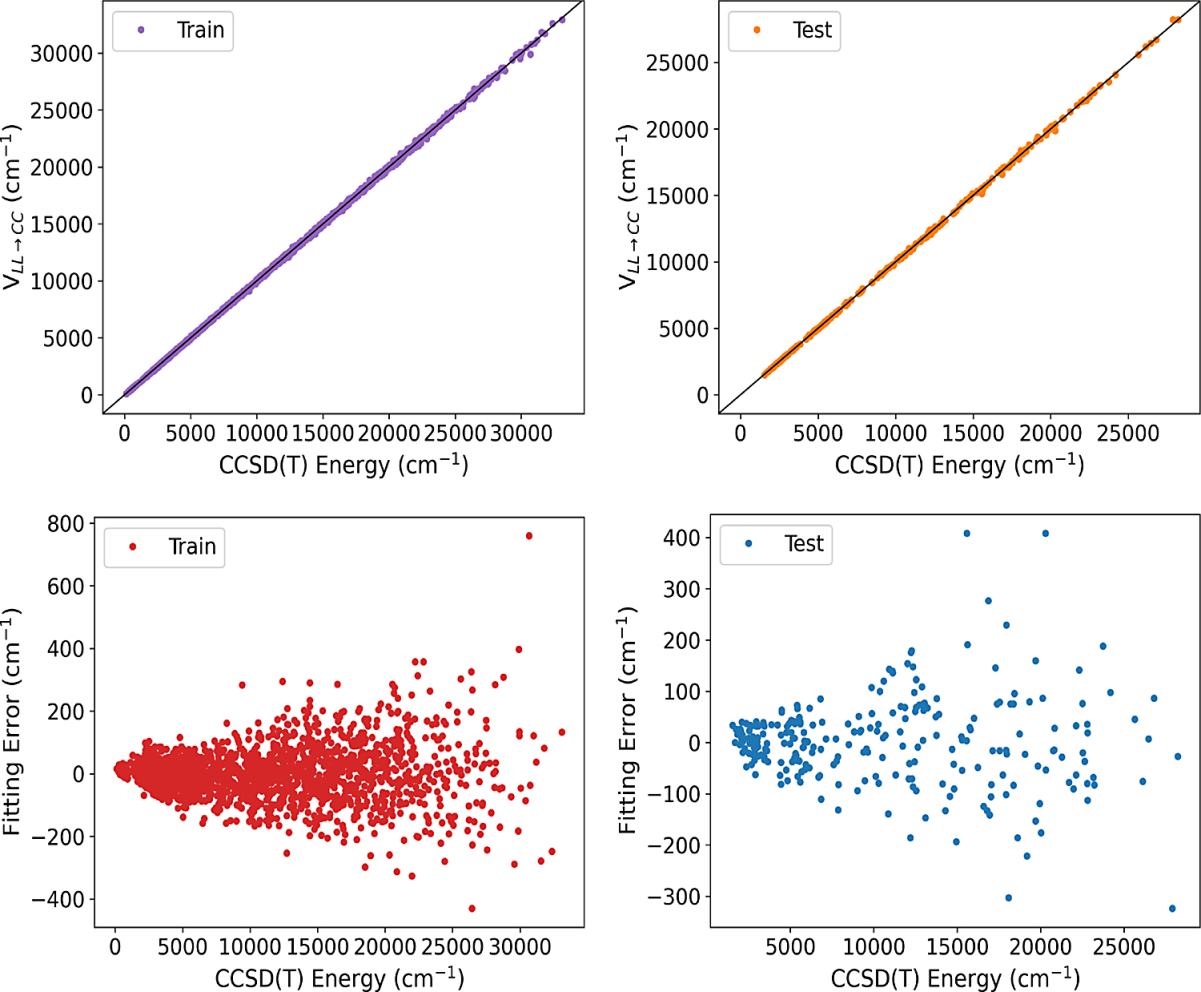
Two upper panels show energies of ethanol from *V*_LL→CC_ vs direct CCSD(*T*) ones for the indicated data sets calculated using the PBE functional. Corresponding fitting errors relative to the minimum energy are given in the lower panels.

Previously, it was noted that the difference between CCSD(*T*) and DFT energies, Δ*V*_CC–LL_, does not vary as strongly as *V*_LL_ with respect to the nuclear configurations and therefore, a small number of high-level electronic energies is adequate to fit the correction PES.

It is not clear if this observation applies, at least semiquantitatively, for classical force fields. In this case, the differences can be much larger, as expected, and indeed verified here for ethanol. We investigate this using a previous data set of 2319 CCSD(*T*)-F12a/aug-cc-pVDZ electronic energies.^49^

As noted above, the permutationally invariant polynomial (PIP) approach is used to fit both the *V*_LL_ and Δ*V*_CC–LL_ PESs. The theory of permutationally invariant polynomial is well established and has been presented in several review articles.^1,2,50−52^ In terms of a PIP basis, the potential energy, *V*, can be written in compact form as
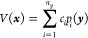
2where *c*_*i*_ are linear coefficients, *p*_*i*_ are PIPs, *n*_p_ is the total number of polynomials for a given maximum polynomial order, and ***y*** are the collection of Morse variables. For example, *y*_αβ_ is given by exp(−*r*_αβ_/λ), where *r*_αβ_ is the internuclear distance between atoms α and β. The range (hyper)parameter, λ, equals 2 Bohr; this is the typical value that has been used in many of our PIPs PESs.^2,50^ The linear coefficients are obtained using standard least-squares methods for large data sets of electronic energies molecules and gradients.

### The Ethanol Force Field

Figure 1 shows conformations of *trans* and *gauche*-ethanol and two saddle point transition states. These are from electronic structure calculations at the CCSD(*T*)-F12a/aug-cc-pVDZ level.

The molecular mechanics force field we consider is the recent one that was corrected using force matching MP2 gradients computed with triple-ζ-quality basis sets using the Adaptive Force Matching method.^41^ The mathematical expression for the total energy of the force field includes intramolecular interaction terms for interactions of atoms that are linked by molecular bonds.
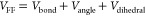
3where *V*_bond_ and *V*_angle_ are modeled by the quadratic energy functions, corresponding to the oscillations about an equilibrium bond length and bond angle, based on the Harmonic approximation, and *V*_dihedral_ is modeled by the cosine function, corresponding to the torsional rotation of four atoms about a central bond.
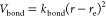
4
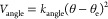
5
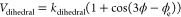
6The value of fitting parameters, *k*_bond_, *k*_angle_, *k*_dihedral_, as well as the equilibrium bond lengths and angles are taken from ref (41).

## Computational Details

We use the data set from our recently reported “MDQM21” data set,^8^ which includes a total of 11,000 energies and their corresponding gradients generated from ab initio molecular dynamics (AIMD) simulations at B3LYP/6-311+G(d,p) level of theory. This data set was partitioned into a training set of 8,500 energies and gradients and a test set of 2500 energies and gradients. The same training and test data set were used for single point energy and gradients computations at M06/6-311+G(d,p),^42^ M06-2*X*/6-311+G(d,p),^42,43^ and PBE/def2-SVP^44^ level of theory using MOLPRO^53^ quantum chemistry package and at PBE0+MBD^45−47^ level of theory with “intermediate” basis setting using the FHI-aims electronic structure package.^48,54^

## Results and Discussion

### Δ-ML for DFT Functionals

The low-level PES, *V*_LL_, is fitted using PIPs with a maximum polynomial order of 4 (14,752 terms) with permutational symmetry 321111. This notation indicates the three equivalent H atoms of the CH_3_- group and the 2 equiv H atoms of the CH_2_ group. The range of the energies for training and testing is 0 to roughly 30,000 cm^–1^. (Note, this energy range is much above the range in the rMD17 data set for ethanol of roughly 8500 cm^–1^.) The root-mean-square (RMS) fitting errors for training and test data sets are shown in Table 1. We see these fits are not overfit and that the precision is high, given the range of the data set.

Next, we train Δ*V*_CC–LL_ on the difference between the CCSD(*T*) and DFT absolute energies at 2069 geometries and test the obtained surface on the remaining 250 geometries. To fit the Δ*V*_LL→CC_, we have used a maximum polynomial order of 2 with permutational symmetry 321111 for the training data set. This results in a basis size of 208 PIPs generated using our MSA software.^55,56^ The RMS training and test errors for the energies of correction PES are shown in Table 2.

**Figure 3:**
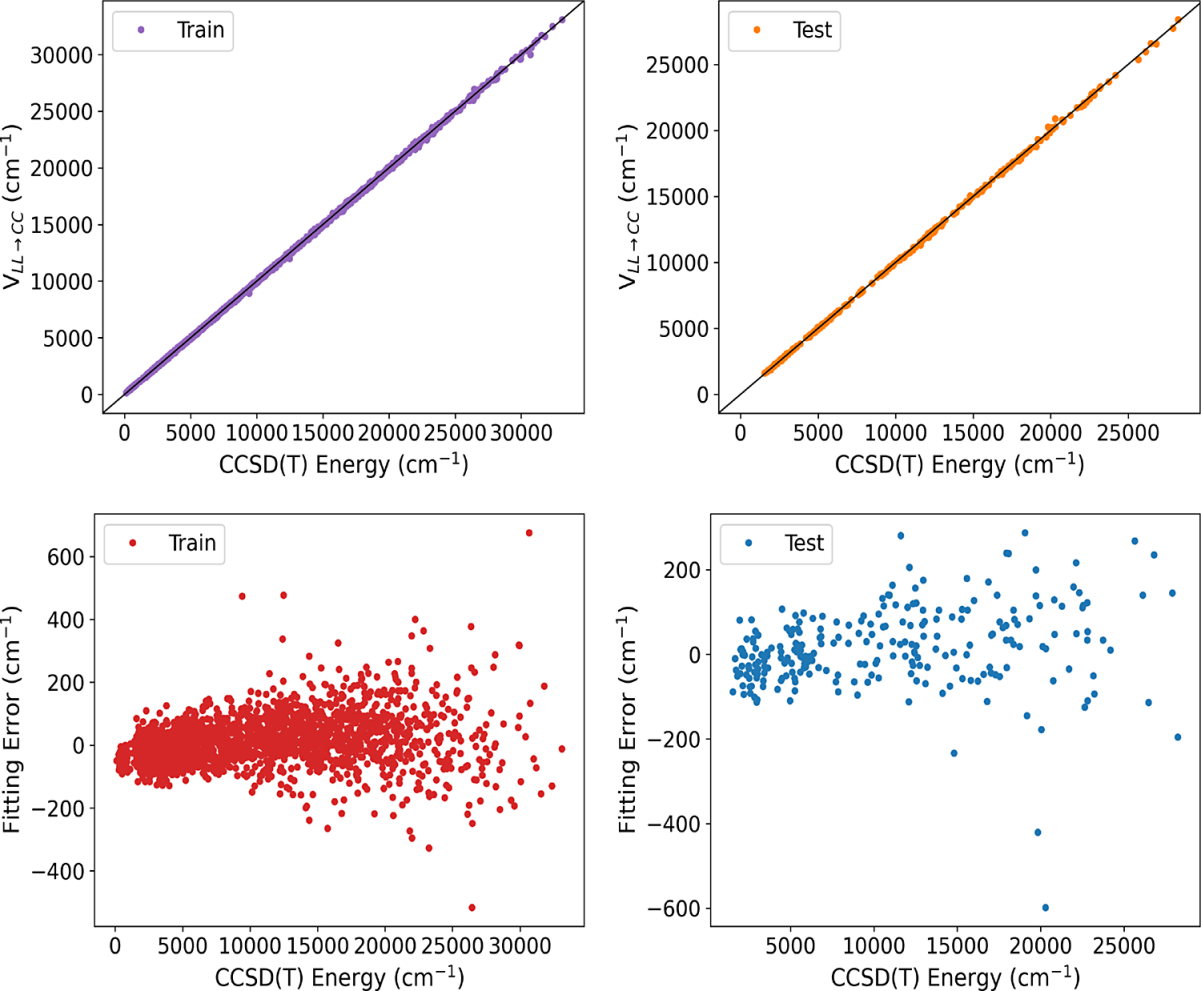
Two upper panels show energies of ethanol from *V*_LL→CC_ vs direct CCSD(*T*) ones for the indicated data sets calculated using the M06 functional. Corresponding fitting errors relative to the minimum energy are given in the lower panels.

Finally, to obtain *V*_LL→CC_ we add the correction Δ*V*_CC–LL_ to the low-level DFT PES, *V*_LL_. The correlation plots of the *V*_LL→CC_ fit for a training set of 2069 points and a test set of 250 points for the PBE and M06 DFT functional are presented in Figures 2 and 3, respectively. The RMS training and test errors for the energies of Δ-corrected PES are shown in Table 3.

**Table 3:** RMS Fitting Error (in cm^–1^) of Corrected PESs *V*_LL→CC_ for Training and Test Data Sets

	PBE	M06	M06-2X	B3LYP	PBE0 + MBD
training	78	79	56	53	53
test	87	97	62	52	52

To determine the accuracy of the *V*_LL→CC_ PES for various DFT functionals, we perform the geometry optimization and normal-mode frequency analysis for both *trans* and *gauche* isomers and their two isomerization saddle point geometries (Anti and Syn). The structures of these isomers and the saddle points are shown in Figure 1. The energetics of all four stationary points of ethanol relative to the trans minima, calculated using various DFT functional, are listed in Table 4. The Δ-corrected PES leads to better optimized energetics for all four stationary points across all DFT functionals, as seen in Table 5.

**Table 4:** Comparison of the Energetics in kcal/mol (cm^–1^) of All Four Stationary Points of Ethanol Relative to the *trans* Minima from Direct DFT Calculations

isomer	PBE	M06	M06-2X	B3LYP	PBE0 + MBD
*trans*	0.00 (0)	0.00 (0)	0.00 (0)	0.00 (0)	0.00 (0)
*gauche*	–0.37 (−129)	0.37 (129)	0.08 (28)	0.05 (18)	0.05 (19)
TS1 (Anti)	1.98 (692)	1.17 (409)	1.16 (407)	1.05 (367)	1.18 (411)
TS2 (Syn)	1.24 (432)	1.69 (591)	1.45 (507)	1.44 (505)	1.13 (395)

**Table 5:** Comparison of the Energetics in kcal/mol (cm^–1^) of All Four Stationary Points of Ethanol Relative to the *trans* Minima for Direct CCSD(*T*) and Δ-ML PESs

isomer	direct CCSD(*T*)	*V*_LL→CC_				
		LL = PBE	LL = M06	LL = M06-2X	LL = B3LYP	LL = PBE0 + MBD
*trans*	0.00 (0)	0.00 (0)	0.00 (0)	0.00 (0)	0.00 (0)	0 (0.00)
*gauche*	0.13 (45)	0.04 (14)	0.21 (73)	0.13 (45)	0.11 (38)	0.14 (51)
TS1 (Anti)	1.09 (381)	1.22 (427)	1.12 (392)	1.08 (378)	1.08 (378)	1.04 (363)
TS2 (Syn)	1.36 (476)	1.41 (493)	1.27 (444)	1.34 (469)	1.35 (472)	1.24 (435)

**Figure 4:**
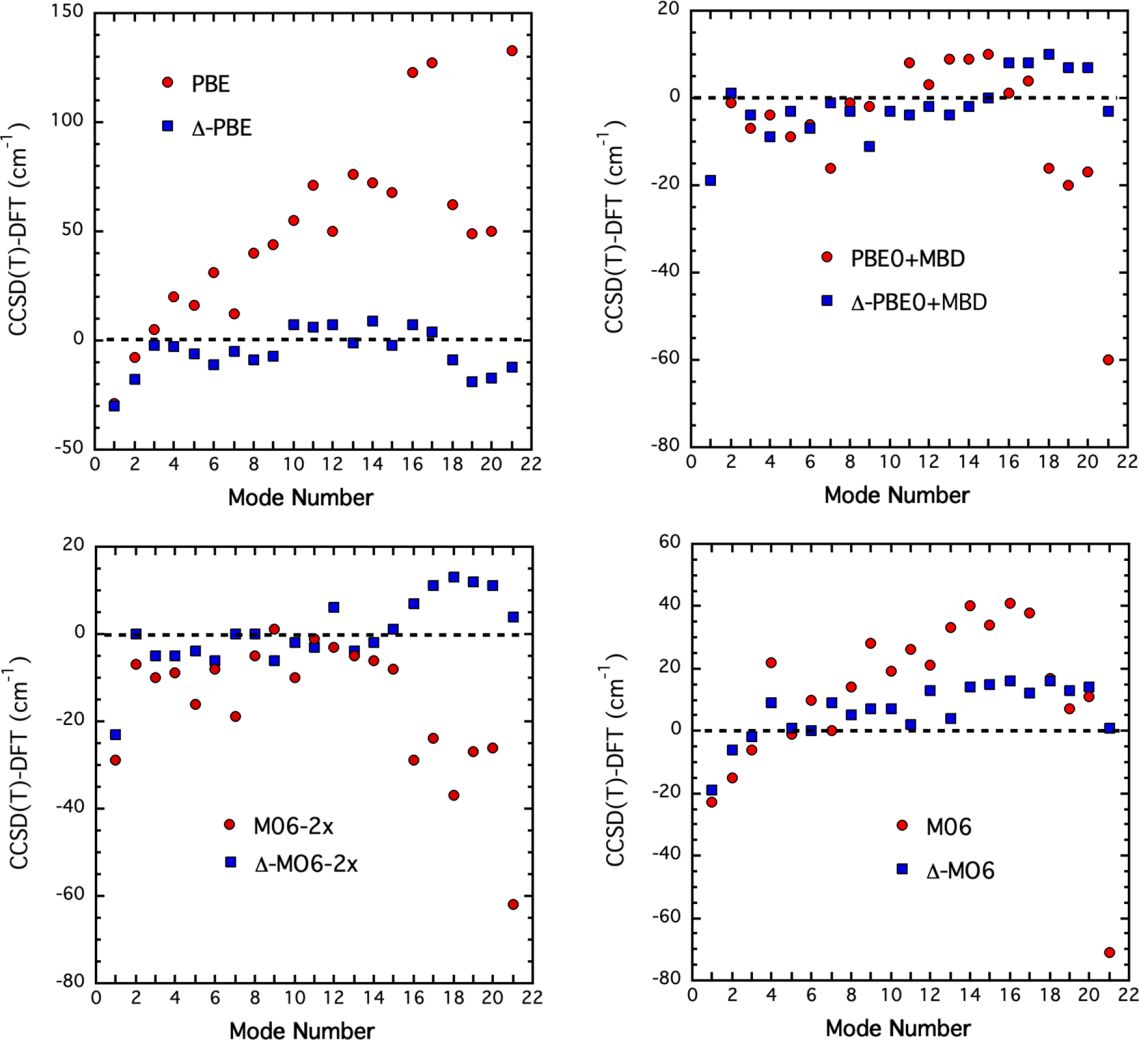
Differences of the CCSD(*T*) and DFT frequencies (in red) and Δ-corrected frequencies (in blue) for *trans*-ethanol for indicated functionals.

The comparison of harmonic mode frequencies of various Δ-corrected PES calculated using different DFT PESs (*V*_LL_) for *trans* ethanol with the corresponding direct CCSD(*T*) frequencies are shown in Table 6. The overall agreement of these harmonic frequencies with the direct CCSD(*T*) ones is excellent, as presented in Figure 4. Note that the Δ-corrected PES tends to minimize the gap between the direct-CCSD(*T*) frequencies and the calculated ones, especially for the high-frequency modes. As depicted in Figure 4, although PBE functional has the highest deviation in frequency from the CCSD(*T*) values, the correction tends to reduce the deviation within a few cm^–1^.

**Table 6:** Comparison of Harmonic Frequencies (in cm^–1^) of DFT PESs and Δ-Corrected PES Computed Using Indicated DFT Functionals and Corresponding Ab Initio Ones (CCSD(*T*)-F12a/aug-cc-pVDZ) for *Trans*-ethanol

mode	CCSD(*T*)	PBE		M06		M06-2X		B3LYP		PBE0 + MBD	
	direct	*V*_LL_	ΔML	*V*_LL_	ΔML	*V*_LL_	ΔML	*V*_LL_	ΔML	*V*_LL_	ΔML
1	222	251	252	245	241	251	245	237	243	241	241
2	274	282	292	289	280	281	274	269	273	275	273
3	413	408	415	419	415	423	418	417	417	420	417
4	813	793	816	791	804	822	818	820	818	817	822
5	907	891	913	908	906	923	911	896	909	916	910
6	1049	1018	1060	1039	1049	1057	1055	1035	1055	1055	1056
7	1115	1103	1120	1115	1106	1134	1115	1094	1115	1131	1116
8	1180	1140	1189	1166	1175	1185	1180	1176	1181	1181	1183
9	1274	1230	1281	1246	1267	1273	1280	1266	1284	1276	1285
10	1300	1245	1293	1281	1293	1310	1302	1299	1302	1303	1303
11	1402	1331	1396	1376	1400	1403	1405	1402	1403	1394	1406
12	1456	1406	1449	1435	1443	1459	1450	1446	1454	1453	1458
13	1484	1408	1485	1451	1480	1489	1488	1483	1488	1475	1488
14	1501	1429	1492	1461	1487	1507	1503	1498	1500	1492	1503
15	1531	1463	1533	1497	1516	1539	1530	1524	1530	1521	1531
16	3001	2878	2994	2960	2985	3030	2994	2978	2995	3000	2993
17	3036	2909	3032	2998	3024	3060	3025	3005	3028	3032	3028
18	3042	2980	3051	3025	3026	3079	3029	3031	3036	3058	3032
19	3122	3073	3141	3115	3109	3149	3110	3098	3120	3142	3115
20	3127	3077	3144	3116	3113	3153	3116	3105	3126	3144	3120
21	3853	3720	3865	3924	3852	3915	3849	3843	3862	3913	3856
MAE		54	9	23	9	16	6	11	4	11	6

**Figure 5:**
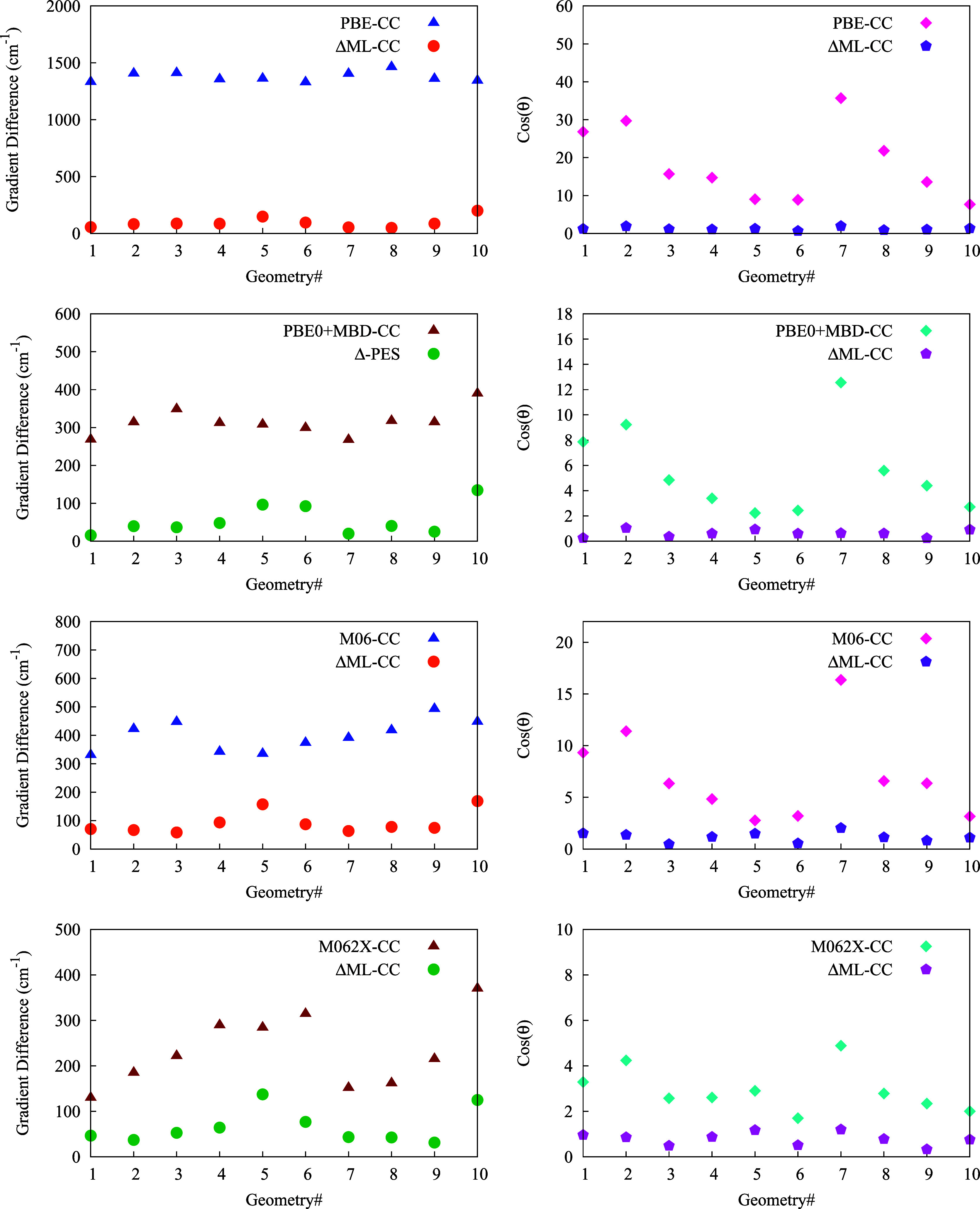
Plot of mean absolute gradient magnitude difference (left panel) and cos θ (right panel), where θ is angle between the direct CCSD(*T*) and indicated DFT gradients as well as corresponding *V*_LL→CC_ PES gradients for randomly selected 10 ethanol geometries. See the text for details.

Next, we examine the change in the PES gradient after the incorporation of the correction. In order to make a more detailed examination of the errors in gradients, we calculated the cosine of the angle between the direct CCSD(*T*) and DFT gradient vector as well as corresponding *V*_LL→CC_ PES gradient vector, also the mean absolute difference (magnitude of 27 gradient components for each geometry) between these two gradient vectors for 10 randomly selected geometries. This is shown in Figure 5. As seen, there is a substantial reduction in the errors in the gradient in the Δ-ML corrected PES compared to the DFT PESs. Specially, in case of PBE the gradient differences are much larger as well as the cos θ values. This is especially encouraging as the correction PES, Δ*V*_CC–LL_, is trained only on CCSD(*T*) energies without CCSD(*T*) gradients. Presumably, including gradient data in the training of Δ*V*_CC–LL_ would result in a larger reduction in the error. We plan to investigate this in the future.

**Figure 6:**
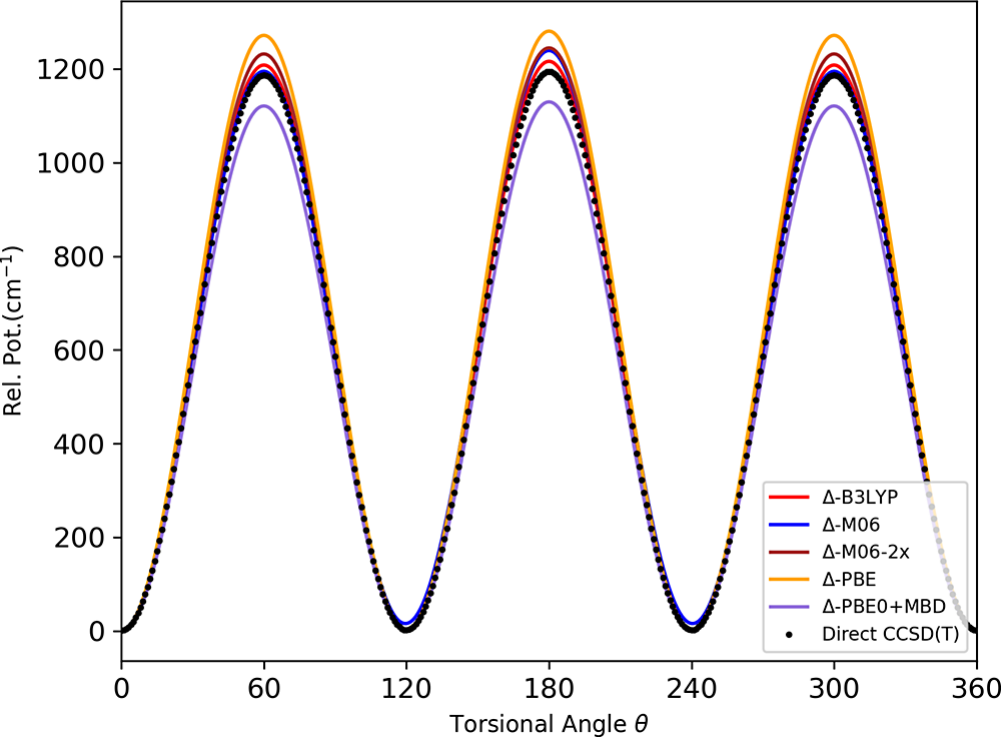
Comparison of torsional potential (not fully relaxed) of the methyl rotor of trans ethanol between direct CCSD(*T*) and Δ-corrected PES computed using indicated DFT functionals.

Next, we compare the PES calculated torsional barrier for the methyl rotor with the direct CCSD(*T*) level. The methyl rotor torsional potentials (not fully relaxed) for the *trans* minima as a function of the torsional angle are shown in Figure 6. For all the DFT functionals, the results from the Δ-corrected PESs are comparable to the direct ab initio calculations at the CCSD(*T*) level, as mentioned in Table 7. Note that the methyl torsional barrier height for the *trans* isomer evaluated from the microwave spectroscopy is 1174 cm^–1^.^57,58^ Similarly, another experimental analysis of the infrared and Raman spectra determined the methyl torsional barriers to be 1185 cm^–1^.^59^

**Table 7:** Barrier Height of the Methyl Rotor Torsional Potential for the Trans Isomer^a^

direct-CCSD(*T*)	Δ-PBE	Δ-M06	Δ-M06-2X	Δ-B3LYP	Δ-PBE0 + MBD
1194	1272	1195	1232	1208	1121

a Energies are in cm^–1^.

To conclude this subsection we comment on the additional cost to evaluate Δ*V*_CC–LL_ relative to the cost to evaluate *V*_LL_. As noted above, the PIPs bases for these two PESs contain 208 and 14,752 terms, respectively. So adding the two potentials results in a negligible 1% increase in cost relative to evaluating *V*_LL_.

### Δ-ML for Force Field

**Figure 7:**
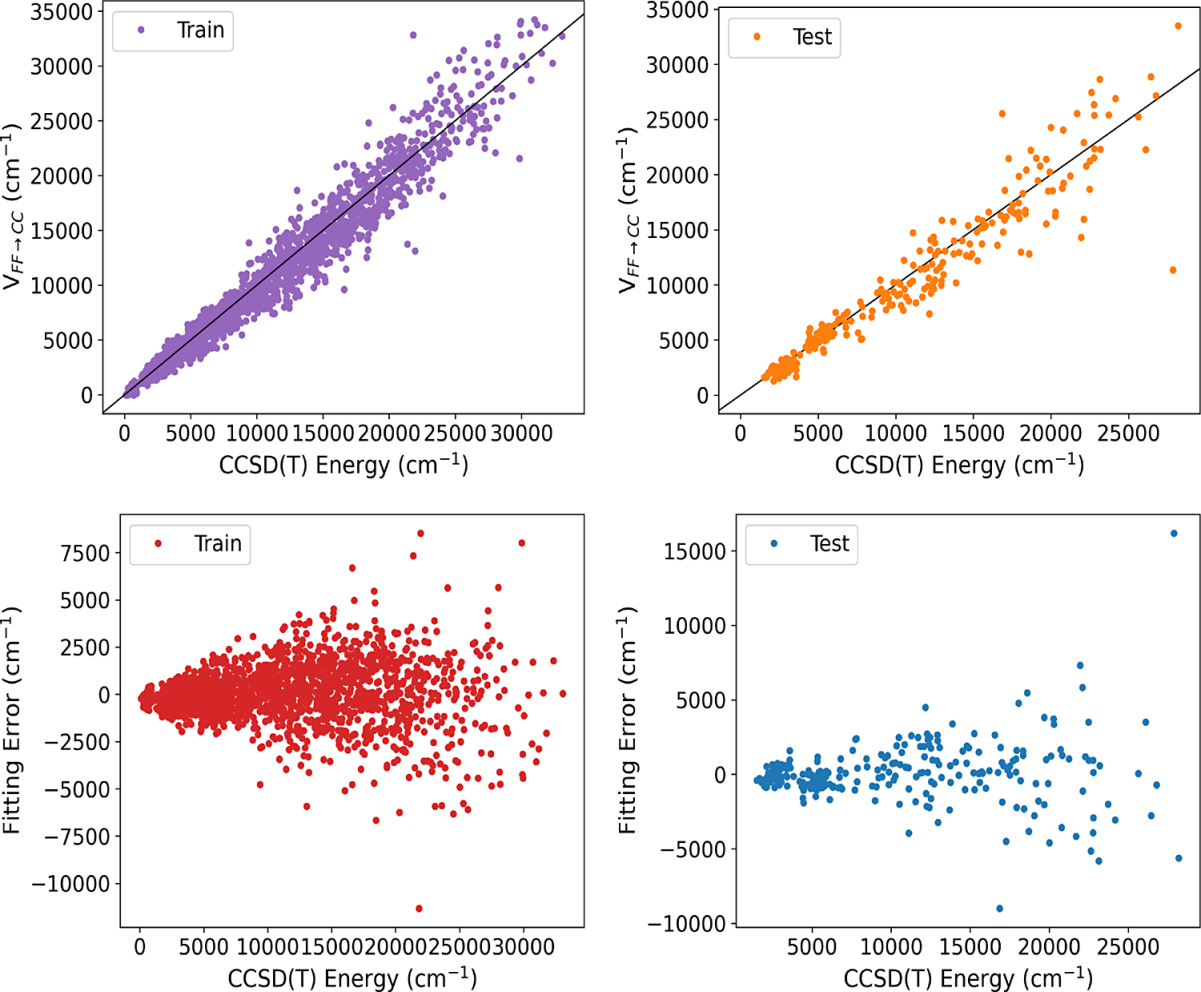
Two upper panels show energies of ethanol from *V*_FF→CC_ vs direct CCSD(*T*) ones for the indicated data sets calculated using the Harmonic approximation for the MP2 corrected force field. Corresponding fitting errors relative to the minimum energy are given in Table 9.

To calculate the Δ-corrected force field potential, we first calculated the force field potential energy using eq 3. Next, we train the Δ-correction PES on the difference between the CCSD(*T*) and FF energies of 2069 geometries and test the obtained surface on the remaining 250 geometries.^49^ To fit the corrected PES, a maximum polynomial order of 2 is used with permutationally symmetry 321111 for the training data set. A plot of V_FF→CC_ versus corresponding direct CCSD(*T*) energies for the training and test sets calculated using the harmonic approximation for the MP2 corrected force field, along with the fitting error, is shown in Figure 7. A huge fitting error for both the training and test sets is found, with RMSE values of 1436 and 2097 cm^–1^, respectively. The substantial RMSE observed in the Δ-ML corrected force field PES indicates the imprecise fitting.

The harmonic approximation works well for the small oscillation around the equilibrium position but its accuracy decreases for larger amplitude vibrations where anharmonicity becomes significant. Hence, we use a Morse potential as *V*_bond_ to provide a more realistic representation to the higher bond stretching.
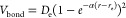
7

Here, the value of α is equal to  for all bond types, with the dissociation energy *D*_e_ provided in Table 8.

**Table 8:** Intramolecular Potential Parameters of Ethanol Taken from Ref (41)

bond type	*r*_e_ (Å)	*k*_bond_ (kcal/mol Å^2^)	*D*_e_ (kcal/mol)
C–C	1.5204	551.9110	82.69
O–H	0.9609	1056.6764	110.66
C–O	1.4396	577.2346	85.56
C–H	1.0937	742.5561	98.71

**Figure 8:**
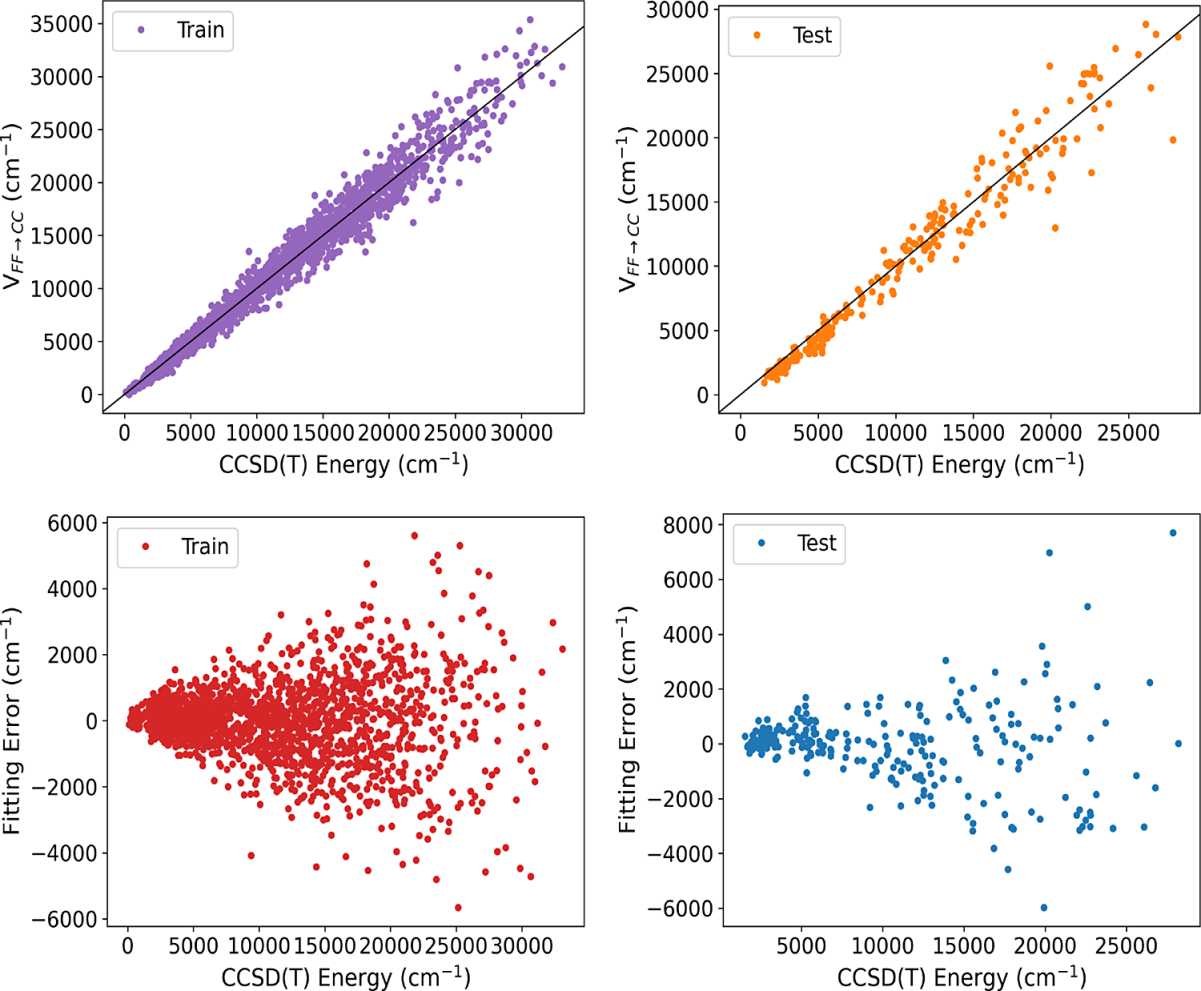
Two upper panels show energies of ethanol from *V*_FF→CC_ vs direct CCSD(*T*) ones for the indicated data sets calculated using the Morse potential for the MP2 corrected force field. Corresponding fitting errors relative to the minimum energy are given in Table 9.

A plot of *V*_FF→CC_ versus corresponding direct CCSD(*T*) energies for the training and test sets, calculated using the Morse potential along with the fitting error, is shown in Figure 8. The fitting error decreases slightly for both the training and test sets compared to Figure 7, with reduced RMSE values of 1089 and 1529 cm^–1^, respectively. However, these RMSE values are still large enough to produce inaccurate results for the entire data set. Therefore, we attempt to improve the fitting by implementing energy cut-offs across the entire data set. For this purpose, we select two energy cut-offs at 10,000 and 5000 cm^–1^ above the global minimum. For the 10,000 cm^–1^ energy cut off case, the correction PES is trained on the difference between the CCSD(*T*) and FF absolute energies of 1124 geometries and tested on the remaining 125 geometries. For the 5000 cm^–1^ energy cut off case, the number of training and test geometries are 702 and 65, respectively.

Table 9 presents the RMS errors in Δ-corrected PES computed using the force field for both the training and test data sets. The RMSE values decrease by a factor of 5 for the energy cutoff set at 10,000 cm^–1^ and by a factor of 15 for the data set at 5000 cm^–1^, compared to the RMSE of the Δ-corrected PES computed using the force field with the harmonic approximation. Since direct CCSD(*T*) energies of the ethanol isomers and their saddle point transition states are quite small in comparison to the RMSE values, their energy optimization results are random. Note that the time taken to calculate 100,000 data points using the force field is 2.04 and 2.09 s for the harmonic and Morse potentials, respectively. Even after adding the Δ-correction, the time taken to calculate 100,000 data points is 2.15 s. Hence, the force field Δ-ML PES is much faster than the one using the DFT functional; to be precise, it has almost doubled the evaluation speed.

**Table 9:** RMS Error in Δ-Corrected Energies (in cm^–1^) Computed Using the Force Field with the Original Harmonic Stretch and Present Morse Potential Modified Stretch Potentials for Train and Test Data Sets^a^

RMS error	Harmonic FF^b^	Morse FF^b^	Morse FF^c^	Morse FF^d^
train	1436	1089	294	98
test	2097	1529	462	233

a These calculations use the fitting basis of 208 terms described in the text.

b Fit using all CCSD(*T*) data to roughly 32,000 cm^–1^.

c Fit using data at 10,000 cm^–1^.

d Fit using data at 5000 cm^–1^.

Next, we performed normal-mode analyses for *trans*-ethanol to examine the vibrational frequency predictions of these PESs. The comparisons of harmonic frequencies with the corresponding ab initio frequencies for the *trans*-ethanol are shown in Table 10. As seen in the table, the original FF produces poor results except for the two lowest frequencies. The sets of corrected FFs all show significant improvement at these frequencies. The corrections to the original FF, denoted as the “Harmonic FF”, and those where the harmonic stretch modes were replaced by Morse potentials, denoted as the “Morse FF”, were evaluated. Overall, the corrected Morse FF results are superior to those of the corrected harmonic FF. Notably, there are interesting dependencies to the extent of the training data set. Limiting the maximum energy to 5000 cm^–1^ produces the best correction, and this is for the Morse FF. This is probably due to the higher precision for the correction PES for this limited energy range, as shown in Table 9. However, the results using the full range still show a significant improvement over the uncorrected FF, with the mean absolute error (MAE) being approximately five times less than that of the uncorrected FF. The reason for this can be deduced from Figures 7 and 8. As seen, the fitting errors are relatively small for energies up to 10,000 cm^–1^ and then grow rapidly above that energy. Therefore, for properties that are largely determined by energies up to 10,000 cm^–1^, such as harmonic frequencies and torsional barriers the correction PES trained on this energy range performs well.

**Table 10:** Comparison of Harmonic Frequencies (in cm^–1^) between *V*_FF→CC_ PES Computed at Indicated Force Field and Corresponding Ab Initio Ones (CCSD(*T*)-F12a/aug-cc-pVDZ) for *Trans*-ethanol

mode	CCSD(*T*)	force field	Harmonic FF	Morse FF		
	direct	harmonic	ΔML^a^	ΔML^a^	ΔML^b^	ΔML^c^
1	222	234	258	381	245	234
2	274	261	276	385	277	240
3	413	606	400	507	351	402
4	813	1131	631	747	748	779
5	907	1148	829	875	917	960
6	1049	1300	835	992	1087	1083
7	1115	1421	1135	1055	1148	1144
8	1180	1443	1320	1090	1165	1191
9	1274	1753	1322	1158	1224	1298
10	1300	1816	1453	1190	1314	1325
11	1402	1960	1481	1195	1347	1413
12	1456	1994	1564	1266	1390	1454
13	1484	2019	1585	1421	1423	1530
14	1501	2049	1600	1480	1427	1539
15	1531	2142	1786	1484	1508	1657
16	3001	4247	1933	3353	3035	3112
17	3036	4300	2200	3442	3224	3135
18	3042	4398	2256	3468	3295	3173
19	3122	4409	2420	3476	3316	3263
20	3127	4410	2674	3502	3322	3304
21	3853	5129	3524	4099	4145	3930
MAE		624	272	116	111	171

a Fit using full data points up to 35,000 cm^–1^.

b Fit using data points up to 10,000 cm^–1^.

c Fit using data points up to 5000 cm^–1^.

**Figure 9:**
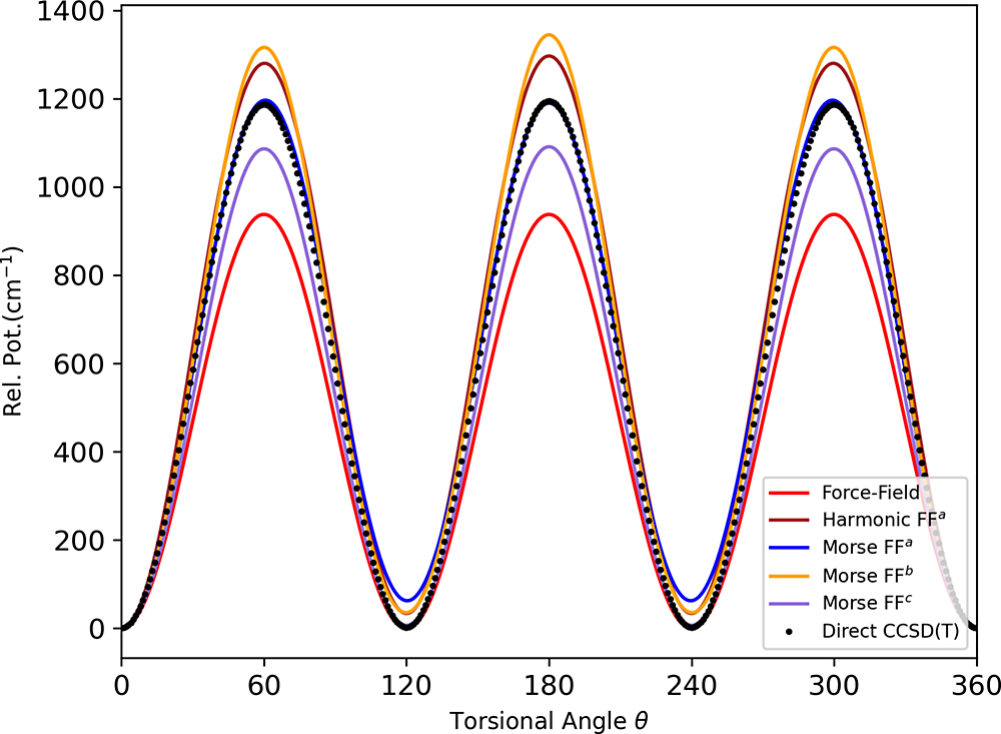
Comparison of torsional potential (not fully relaxed) of the methyl rotor of trans-ethanol between direct CCSD(*T*) and Δ-corrected PES computed using the force field.

Lastly, we analyzed the torsional barrier for the methyl rotor calculated by Δ-ML PES using the force field. The results of the methyl torsional barrier height for the trans isomer, calculated from the various PESs of the force field, are listed in Table 11. As shown in Figure 9, the torsional barrier height for the harmonic force field is much lower than the direct CCSD(*T*) value. For all the corrected PESs, the barrier height improves. The barrier height matches to the direct CCSD(*T*) value for the Morse FF when the full data set is considered.

**Table 11:** Barrier Height of the Methyl Rotor Torsional Potential Calculated Using Δ-Corrected Force Field for the Trans Isomer^a^

CCSD(*T*)	force field	Harmonic FF	Morse FF		
direct	harmonic	ΔML^b^	ΔML^b^	ΔML^c^	ΔML^d^
1194	938	1280	1194	1316	1086

a Energies are in cm^–1^.

b Fit using full data points up to 35,000 cm^–1^.

c Fit using data points up to 10,000 cm^–1^.

d Fit using data points up to 5000 cm^–1^.

Overall, the correction to this classical FF has been successful. And, it is reasonable to ask how the approach taken could be used for general classical FFs, especially for molecules much larger than ethanol. There is not a simple answer to this question, but clearly this is a fruitful area for future work. One preliminary thought is to make use of the simple form of FFs, which is just the generalization of eqs 4–6, and to correct groups of terms instead of the entire FF.

We end this section with some remarks about the history of dual-level (broadly defined) approaches to developing ab initio-based PESs. Perhaps one of the earliest examples dates to 1985, where we would consider a low-level ab initio quartic force field for formaldehyde was reported. In this instance, the harmonic force constants were adjusted to improve and agree with the experiment using vibrational configuration interaction calculations, with all other force constants unchanged.^60^ This early approach evolved into a more sophisticated one using the *N*-mode representation of the potential.^61^ By using this representation, different levels of electronic structure theory are used for the different n-mode coupling terms.^62−65^

For global potentials, the Shepard interpolation method of Collins^66^ was extended for chemical reactions using a dual-level method.^67^ Specifically, high-level data points are placed along the reaction path, whereas the rest of the configuration space is described by a low-level method.

More recent work using machine learning to correct PESs uses Transfer Learning (TL)^68,69^ and the Δ-ML described above. In TL the nonlinear parameters of a model, typically a neural network, trained on low-level data are retrained on sparse high-level data. This approach has been used successfully and extensively by Meuwly and co-workers for a number of applications, including anharmonic vibrational analyses, chemical reaction dynamics, and tunneling splittings.^70−73^

For the present approach, i.e., eq 1, we note that expression has been used to develop reaction PIP-NN PESs.^74,75^ In this approach the correction potential Δ*V*_CC–LL_ is fit using the PIP-NN method,^76^ and then used to generate data at the configurations where the low-level calculations were done. Then the sum of the low-level energies and those from Δ*V*_CC–LL_ are fit, again using the PIP-NN method. This results in a single Δ-corrected PES in contrast to the approach used here where the Δ*V*_CC–LL_ and the low-level PES are added together. We examined these two approaches for a PIP potential for the formic acid-ammonia dimer.^33^ As expected, they yield virtually identical results. As already noted, the additional overhead in using the two-PES approach is small or, as in the present case, negligible compared to the one-PES. So, based on this alone, there is not a strong reason to prefer one approach over the other. However, we do suggest reporting the correction PES Δ*V*_CC–LL_ whichever approach is used. This will be useful in the event that holes are found in *V*_LL→CC_ or the single PES, say in high energy dynamics calculations.

## Summary and Conclusions

The generality of the single-step Δ-ML method we proposed and applied using B3LYP to a number of PIP PESs has been demonstrated here for ethanol using other popular DFT functionals. In each case, the Δ-ML method produces a substantial improvement in accuracy compared to the CCSD(*T*) benchmark results. The most dramatic improvement is observed in the harmonic frequencies, where the DFT PIP PESs produce both significant underestimates and overestimates of the CH and OH-stretch frequencies. Additionally, we achieved significant improvement over DFT gradients without using CC gradient data to correct the PES. An exploratory application of this Δ-ML method to a recent force field (FF) for ethanol was given. Notably, the inaccurate harmonic frequencies at the global minimum from the force field are significantly corrected. The torsional barrier from the FF is also improved using the Δ-ML method. Additionally, the computational cost for the correction is about the same as the cost to evaluate the simple FF.

The new DFT Δ-ML potentials are expected to perform as well for Diffusion Monte Carlo and VSCF/VCI calculations as the original B3LYP Δ-ML one.^49,77^ (We remind the interested reader that this corrected PES is available in Supporting Information in ref (77).) However, the performance of the Δ-ML corrected Force Field will need to be investigated for such calculations. In addition, it will also be of interest to test the new Quantum-Monte Carlo based sGDML potential^78^ for such calculations.

Finally, this one-step Δ-ML method is very straightforward and can be easily implemented into other ML methods or descriptors. While ethanol molecule is used here as a prototype example, this approach is also applicable to large molecular systems for developing machine-learned force fields as accurate as the CC level.

## Abbreviations

PES: potential energy surface
